# Predicting enzyme targets for cancer drugs by profiling human Metabolic reactions in NCI-60 cell lines

**DOI:** 10.1186/1471-2105-11-501

**Published:** 2010-10-08

**Authors:** Limin Li, Xiaobo Zhou, Wai-Ki Ching, Ping Wang

**Affiliations:** 1 Institute of Information and System Science, Xi'an Jiaotong University, Xi'an, 710049, China; 2 Center for Biotechnology and Informatics, The Methodist Hospital Research Institute and Department of Radiology, The Methodist Hospital, Weill Cornell Medical College, Houston, TX 77030, USA; 3 Advanced Modeling and Applied Computing Laboratory, Department of Mathematics, The University of Hong Kong, Pokfulam Road, Hong Kong; 4 The Methodist Hospital Research Institute and Department of Pathology, The Methodist Hospital, Weill Cornell Medical College, Houston, TX 77030, USA

## Abstract

**Background:**

Drugs can influence the whole metabolic system by targeting enzymes which catalyze metabolic reactions. The existence of interactions between drugs and metabolic reactions suggests a potential way to discover drug targets.

**Results:**

In this paper, we present a computational method to predict new targets for approved anti-cancer drugs by exploring drug-reaction interactions. We construct a Drug-Reaction Network to provide a global view of drug-reaction interactions and drug-pathway interactions. The recent reconstruction of the human metabolic network and development of flux analysis approaches make it possible to predict each metabolic reaction's cell line-specific flux state based on the cell line-specific gene expressions. We first profile each reaction by its flux states in NCI-60 cancer cell lines, and then propose a kernel k-nearest neighbor model to predict related metabolic reactions and enzyme targets for approved cancer drugs. We also integrate the target structure data with reaction flux profiles to predict drug targets and the area under curves can reach 0.92.

**Conclusions:**

The cross validations using the methods with and without metabolic network indicate that the former method is significantly better than the latter. Further experiments show the synergism of reaction flux profiles and target structure for drug target prediction. It also implies the significant contribution of metabolic network to predict drug targets. Finally, we apply our method to predict new reactions and possible enzyme targets for cancer drugs.

## Background

The dysregulation of the Human metabolism is closely related to human diseases. Many human diseases are metabolic diseases, which are directly caused by a deficiency of metabolic enzymes and the subsequent accumulation of toxic substances or the lack of essential metabolites. Many common diseases such as cardiovascular disease, cancer and diabetes are related to a malfunction of the human metabolism. The statistics from OMIM show that around 23% of the metabolic genes are disease-related genes and more than 48% of the metabolic reactions are affected by disease-related genes [1]. The emergence of metabolic diseases has stimulated research in human metabolism and its regulation to discover the mechanism of drug-target interactions. Enzymes catalyze reactions, which produce metabolites in the metabolic network of an organism. Enzyme malfunctions can cause the accumulation of certain compounds that may result in diseases. In fact, many enzymes have already been selected as drug targets [2,3]. An effective drug may target the enzymes to recover the concentration of signal metabolites that are in abnormal states. Thus, it is important for drug development to identify more enzyme targets.

Recently, significant work has been gradually done to reconstruct human genome-scale metabolic network, which provides a unified platform to integrate the experimental data for genes, proteins, drugs, drug targets, metabolites and metabolic reactions in the same system for the study of human metabolism and the identification of novel drug targets. Currently there are two high quality human metabolic networks developed independently: EHMN reconstructed by Ma et al. [4] and Human Recon 1 reconstructed by Duarte et al. [5]. Note that Human Recon 1 has been adapted for flux analysis while EHMN has not. In our study, we use Human Recon 1 since our method is based on flux analysis. A metabolic network consists of three parts of metabolic information: 1) stoichiometry; 2) the relationships between enzymes and metabolic reactions; and 3) flux capability of each metabolic reaction. Stoichiometry provides the quantitative relationships between reactants and products for all metabolic reactions, and the relationships between enzymes and metabolic reactions identify enzymes that catalyze each reaction. In the Human Recon 1 metabolic network, 2299 (61%) of the total 3742 metabolic reactions are catalyzed by enzymes, which we call enzymatic reactions.

One traditional approach to analyze a metabolic network is constraint-based flux distribution prediction, which employs stoichiometric, thermodynamic, flux capability and other constraints to determine the space of flux distributions. One commonly used constraint-based approach is Flux Balance Analysis (FBA), which utilizes stoichiometries to predict a network's ability to produce pseudo-steady state conditions[6]. Recently, Shlomi et al. [7] proposed a computational constraint-based method for systematically predicting human tissue-specific metabolic behavior in ten human normal tissues by integrating the human metabolic network with tissue-specific gene and protein-expression data. Although their work was focused on gene activity prediction in a specific tissue, their method provided a way to connect the states of metabolic reactions in the metabolic network with specific cellular environments. This motivates us to profile each metabolic reaction by analyzing its fluxes in different cellular environments such as NCI-60 cancer cell lines. Thus, each reaction can be represented as its reaction fluxes in the different cells. This is the reaction representation method what we call reaction profiling. By this method, we can construct the flux similarity between the reactions and then further use the machine learning methods to analyze their properties in the metabolic network.

A promising approach in drug target discovery involves the integration of available biomedical data through mathematical modeling and data mining. Significant work [8-11] has been done on drug discovery by integrating different data sources including gene expression data, protein sequence, mass spectrum, chemical structure, drug response and side effects. However, few papers discussed the integration of these data with the human metabolic network. Almaas et al. [12] applied flux-balance analysis to identify a set of metabolic reactions forming a connected metabolic core and found that the enzymes catalyzing the core reactions display a considerable higher fraction of phenotypic essentiality and evolutionary conservation than those catalyzing noncore reactions. This implies the importance of understanding the properties of metabolic reactions. They also found that most current antibiotics interfering bacterial metabolism target the core enzymes and implied that metabolic reactions play an important role in drug-target discovery. Sridhar et al. [13,14] formulated an optimal enzyme combination identification problem on metabolic networks, but the focus was to select a subset of enzymes that can reduce the amount abnormally accumulated metabolites. In this paper, we proposed a supervised method by integrating the human metabolic network and gene expression to predict new related reactions for old drugs. We first constructed several similarity measurements for enzymatic reactions (flux similarity mentioned above and structure similarity discussed later), and then applied machine learning approaches to predict novel drug-reaction interactions based on their known interactions, which are obtained from known drug-target interactions. We also constructed a Drug-Reaction Network (DRN) based on these known interactions to get a deeper understanding of the influences of drugs on the metabolic network. Thus our drug-target prediction procedure is mainly that we first did reaction profiling and meanwhile extracted known drug-reaction interactions, and then used machine learning approaches to predict new drug-reaction interactions. There are two innovations in our method. The first is that we constructed the DRN and predicted drug targets by predicting the interactions between drugs and metabolic reactions. Drugs are considered to influence several enzymatic reactions, since they can interact with enzymatic reactions through their targets as enzymes. Thus, a bipartite network between drugs and enzymatic metabolic reactions (named as Drug-Reaction Network) can be constructed. This network shows the influences of drugs on the metabolic reactions and the metabolic pathways from a global view. The second innovation of our method is the new concept of reaction profiling for each metabolic reaction and the new measurement for reaction similarity in the context of a specific disease, such as cancer. By this method, each reaction can be represented quantitatively as its flux states in NCI-60 cell lines, and thus computational models can be used to predict novel drug-reaction interactions for cancer drugs, which leads to the discovery of their novel drug targets. We also further improve the performance by integrating target sequence data with the reaction profiling, and the results show the synergism of structural similarity and functional similarity between reactions for drug reaction/target interaction prediction.

## Methods

### Data and softwares

The genome-scale human metabolic network reconstructed by Duarte et al.[5] consists of 2766 metabolites, 3742 reactions and 1905 genes. We downloaded the data from the BiGG database http://bigg.ucsd.edu/. The COBRA toolbox [15] developed by Palsson's group was then used to parse the data. Gene expression data (mRNA:Affy-U133B, GCRMA-normalized) in NCI-60 cell lines conducted by Shankavaram et al. [16] was downloaded from NCI website. In fact, only 59 cell lines have available gene expression data. We also downloaded protein sequence data from KEGG database [17], and computed the protein sequence similarities using Smith-Waterman method by Matlab. Drug-target association data was downloaded from DrugBank. In the process of constraint-based flux analysis, we used the freely available software, GLPK solver, to solve LP/MILP problems. In the following subsections, we will discuss the details of the two models: The Linear Programming (LP) Model for flux analysis and The Kernel KNN Model for drug-reaction interaction prediction.

### Linear programming model for flux analysis in NCI-60 cell lines

A network-based method is proposed by Shlomi et al. [7] to predict human tissue-specific metabolic behavior in ten tissues by integrating tissue-specific gene and protein expression data with the global human metabolic network, and their work reveals a central role for post-transcriptional regulation in shaping tissue-specific metabolic activity profiles. This inspires us to analyze cell line-specific metabolism based on gene expression data in NCI-60 cancer cell lines. By integrating the metabolic network and the cell line-specific gene expression, we can predict reaction flux and activity for all the reactions in each cell line by our proposed linear programming model. Thus, each metabolic reaction in the metabolic network has 59 flux levels in 59 cell lines. Reaction profiling is the procedure to represent each reaction as its flux values in NCI-60 cell lines.

For a specific cell line, we first identify highly/lowly expressed genes based on the gene expression data in this cell line. Then for each metabolic reaction, if one of its enzyme genes is highly expressed, the reaction is considered as highly expressed. If all of its enzyme genes are lowly expressed or undetermined, the reaction is considered as lowly expressed or undetermined, respectively. The boolean gene-to-reaction mapping can be obtained from the metabolic network model. Thus, based on the expression states of the corresponding genes, all the reactions are classified into a highly expressed subset *R*_*H*_, a lowly expressed subset *R*_*L *_and an undetermined subset. We revised Shlomi's Mixed Integer Linear Programming (MILP) model to a Linear Programming (LP) model by relaxing the integer variables *y*_i_^+ ^and *y*_i_^- ^to be continuous. The reasons for the relaxation are twofold. First, the relaxation allows the activity score of reactions to be continuous rather than binary value, which is more reasonable and flexible. Second, LP model is much easier to handle than MILP model. The LP model is presented as follows:

s.t.

where **v **is the flux vector in the considered specific cell line for all reactions, **S **is the stoichiometric matrix obtained from the metabolic network, and *ε *is a flux threshold. The flux threshold is to control the distribution of the likelihoods *y*_i_^+ ^and *y*_i_^- ^from the constraints of  and . A highly expressed reaction is considered to be more likely to be active if it carries a significant positive flux that is greater than a positive threshold *ε *or a significant negative flux < -*ε*. Lowly expressed reactions are considered to be more likely to be inactive if they carry almost zero metabolic flux. Flux threshold *ε = *1 is used throughout the experimental analysis although its changes did not make a big difference in the results. For a highly expressed reaction *i*, *y*_i_^+ ^and *y*_i_^- ^are the likelihood that reaction *i *is active in either direction. Their closeness to one means *i *is likely to be active, while the closeness to zero means it is likely to be inactive. For a lowly expressed reaction *i*, *y*_i_^+ ^represents the likelihood that it is inactive. We first apply LP model in each of NCI-60 cancer cell lines to predict flux distribution for all the metabolic reactions, and then, we can obtain for each reaction its flux profile in these 59 cancer cell lines.

### Reaction flux similarity

We denote the set of considered reactions as *R*, and define Γ_*RDR *_and Γ_*RER *_as follows: Γ_*RDR/RER *_= {(*i*, *j*) ∈ *R *× *R*| *i*, *j *are associated with same Drugs/Enzymes}. Γ_*RDR *_includes all reaction pairs that are associated with the same drugs, while Γ_*RER *_includes all reaction pairs associated with the same enzymes. Based on any reaction similarity metric *s*, we can define the conditional probability:

where |S| is the number of elements in set **S**, and *s*(*i*, *j*) is the similarity between reaction *i *and *j*. *P*_*E*_(*t*) is defined in the same way. *P*_*D*_(*t*)/*P*_*E*_(*t*) are conditional probabilities that a pair of reactions share the same drugs/enzymes if their similarity score is larger than *t*. An ideal similarity metric *s *makes pairs of reactions with larger similarity score share the same drugs/enzyme with higher probability. This means the *s*-based conditional probabilities *P*_*D*_(*t*)/*P*_*E*_(*t*) should be increasing functions of *t*.

We now define a similarity metric based on reaction flux profiles and then check whether it's a possible ideal metric. The flux of reaction *i *in *j*th cell line, *f*_*ij*_, can be obtained from the optimal solution **v **of the above linear programming model for *j*th cell line. We represent the profile for reaction *i *as **f**_*i*_ = [*f*_*i*1_, ...,*f*_*i*j_,...,*f*_*i*,59_]^*T*^. Each reaction is thus profiled using its 59 flux values in NCI-60 cell lines, and can be considered as a data point in 59-dimensional flux feature space. We use cosine similarity (discussed more in Additional file 1) of reaction flux profiles to measure the similarity among metabolic reactions: . This is called reaction flux (RF) similarity between reaction *i *and *j*. It turns out in the results section that, s_*RF*_-based probabilities *P*_*D*_(*t*)/*P*_*E*_(*t*) are increasing with *t*, thus the similarity metric s_*RF *_is likely to be an ideal metric for drug-reaction interaction prediction.

### Reaction structure similarity

Denote the set of target enzymes for reaction *i *by *M*_*i*_. Target sequence (TS) similarity for protein *s *and *t *is denoted by s_*TS *_(s,*t*) (normalized to be between 0 and 1). Then the reaction similarity for reaction *i *and *j *based on target sequence is defined as the maximum, average or minimum of all the possible enzyme target sequence similarities , , and . For simplicity, this is called reaction structure (RS) similarity s_*RS *_since it is defined based on enzyme targets' structure.

s_*RF *_measures the functional similarities among reactions, while s_*RS *_measures their similarity based on enzyme targets' structural information. The integration of s_*RF *_and s_*RS *_may provide an optimal measurement. Thus we define a new reaction similarity s_*R *_as the weighted sum of reaction flux similarity and reaction structure similarity: s_*R *_(*i*, *j*)=*λs*_*RF *_(*i*, *j*) + (1 - *λ*) *s*_*RS*_(*i*, *j*) for any reaction *i *and *j*.

### Kernel KNN model

For a specific drug, we can obtain its true interactions with *m *reactions from known drug-target interactions. We aim to predict whether a new metabolic reaction interacts with the drug or not. Here we propose a Kernel KNN (K-Nearest Neighborhood) method for this binary classification problem using the similarities between reactions. We denote *x*_*i *_= 1 if the drug has interaction with the reaction *i*, *x*_*i *_= 0 otherwise, for *i *= 1,...,*m*. Then, for a new metabolic reaction, we predict its interaction with all drugs as , where *s *is a reaction similarity metric, *s*(*new*, *i*) represents the similarity between the new reaction and reaction *i *and *N*{*k*} is the set of *k *reactions nearest to the new reaction under the meaning of the metric *s*. *s *can be s_*RF*_, s_*RS *_or their integration s_*R*_. Here *x*_*new *_is called the association score of the new reaction and the specific drug. Higher association score indicates a higher probability of the reaction interacting with the drug. Kernel KNN is a kernel version of KNN classification method. Note we still call this model as Kernel KNN method even if *s *may not be positive semi-definite. Both KNN and Kernel KNN classify a new point simply through its nearest neighborhood. However, Kernel KNN considers the weight of voting from different individuals in the neighborhood.

## Results and discussion

### Drug-Reaction Network construction

**Figure 1:**
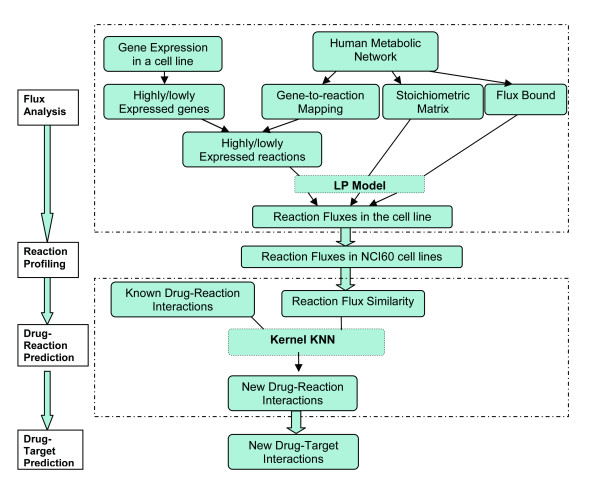
**Flow chart for drug-target prediction**. The 4-stage task includes flux analysis, reaction profiling, drug-reaction prediction and finally drug target prediction.

The flow chart of the whole task of drug reaction/target prediction using reaction flux similarity is shown in Figure 1. This task involves four stages including flux analysis, reaction profiling, drug-reaction interaction prediction and drug-target prediction. In the first stage, metabolic reactions in the metabolic network are first classified as highly or lowly expressed based on the expression levels of the genes which encode the enzymes catalyzing the reactions. Integrating the classified reactions with metabolic stoichiometry, the LP model is used to predict the flux distribution for metabolic reactions in the different cellular environments of 59 cancer cell lines. After generating reaction profiles in stage two by putting reaction fluxes of 59 cell lines together, reaction flux similarity is constructed to measure the similarities among reactions functionally. With reaction flux similarity and their known interactions with drugs, stage three applies the proposed Kernel KNN model to predict novel drug-reaction interactions. The last stage is to obtain novel drug targets using the hints from drug-reaction interactions. In this section, we discussed how to construct the Drug-Reaction Network based on the known drug-target interactions, and then reported some results from the analysis of this network.

**Figure 2:**
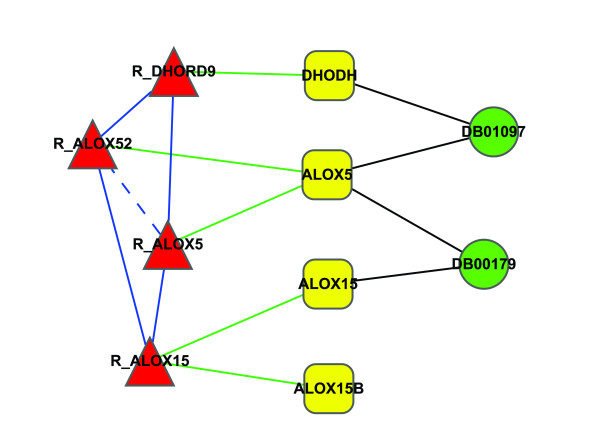
**An illustrative example of the relationship between drugs, enzyme targets and metabolic reactions**. Green circle, yellow rectangle and red triangle represent drug, protein and reaction, respectively. Black edge and green edge indicate drug-target interaction and reaction-enzyme relationship. The blue solid edge between two reactions indicates that two reactions interact with the same drug through different enzyme target, while blue dashed edge means that two reactions interact with the same drug through the same enzyme targets.

Although relationships between drugs and targets have been depicted in a global view [18], the relationship between drug and enzymatic reactions remains uncharacterized. The effect of a drug on human metabolism takes place through its target protein, which, as an enzyme, can catalyze its corresponding metabolic reactions directly. Thus, if any target of a drug catalyzes a reaction as an enzyme, the drug can interact with the reaction through the enzyme target and the reaction is considered to be a target reaction of the drug. An illustrative example is shown in Figure 2. The green circle, yellow rectangle and red triangle represent drug, protein and reaction, respectively. Black edges and green edges indicate drug-target relationships and reaction-enzyme relationships. We consider two approved anti-cancer drugs DB01097 (Leflunomide) and DB00179 (Masoprocol). From the Figure 2, we can see that DB01097 interacts with reaction R_DHORD9 through the enzyme target DHODH, and DB00179 interacts with R_ALOX15 through the enzyme target ALOX15. Both drugs interact with R_ALOX52 and R_AL0X5 through ALOX5. The blue solid edge between two reactions in Figure 2 indicates that two reactions interact with the same drug through different enzyme targets, while the blue dashed edge means that two reactions interact with the same drug through the same enzyme targets.

**Figure 3:**
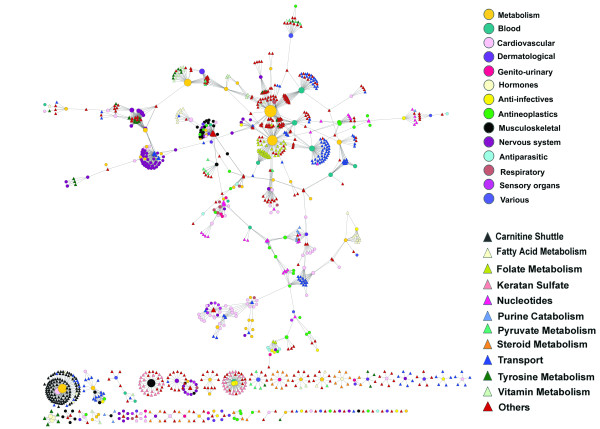
**Drug-Reaction Network (DRN)**. DRN is generated by using the known interactions between approved drugs and their target reactions. Circles and triangles correspond to drugs and reactions, respectively. An edge between a drug node and a reaction node is placed if the drug targets at least one enzyme of the reaction. The area of the drug (reaction) node is proportional to the number of reactions (drugs) the drug (reaction) interacts with. Drug nodes are colored according to their Anatomical Therapeutic Chemical Classification, and reactions are colored according to their subsystems obtained from human metabolic network data.

**Figure 4:**
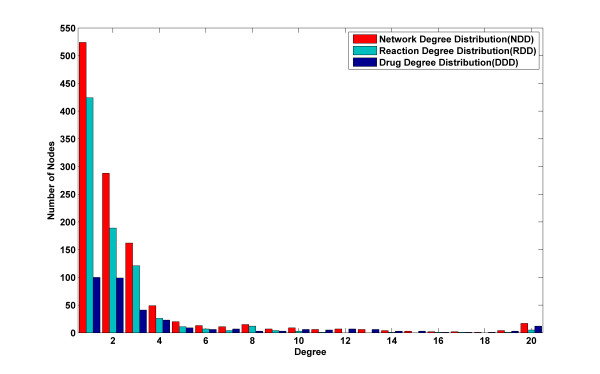
**Degree distribution in Drug-Reaction Network: Red represents the histogram for all nodes in DRN**. Green and blue represent the histograms for drug nodes and reaction nodes, respectively.

We constructed a Drug-Reaction Network (DRN) to depict the interactions between the approved drugs and metabolic reactions. We used Cytoscape [19] to show the network in Figure 3. The nodes in DRN include drugs and enzymatic reactions, and the edges between drugs and reactions indicate their interactions. Two kinds of data are required to construct this bipartite graph: drug-target interaction data, which was obtained from the DrugBank [20] database, and gene-to-reaction mapping data, which was obtained from metabolic network Human Recon 1 reconstructed by Duarte et al. [5]. All drug-reaction interactions and the corresponding enzyme targets are listed in Additional file 2, Table S1. The resulting Drug-Reaction Network includes 339 (23%) of 1492 whole approved drugs, 811 (35%) of 2299 enzymatic metabolic reactions, 338 enzyme targets and 1800 interactions between drugs and reactions. Figure 4 shows the degree distribution in DRN. We report the numbers of nodes (both drugs and reactions, reactions only and drugs only) with different degrees from 1 to 20, which are the network degree distribution, reaction degree distribution and drug degree distribution, respectively. There are 80% of the drugs and 95% of the reactions in DRN with a degree less than 5. Maximal degree of the drugs and reactions are 112 and 35, respectively.

We also colored all the drug nodes in Figure 3 according to the Anatomical Therapeutic Chemical (ATC) Classification of drugs. Detailed classification information for each drug and statistics in DRN is shown in Additional file 2, Table S2 and Table S3, respectively). We found that around half of the approved drugs in DRN are for metabolic diseases, cardiovascular diseases and antineoplastic drugs. This implies that among all 14 classes of drugs, these three classes are related to metabolic network most closely. We also found that DRN has 29 (50% of total) approved drugs for Musculoskeletal diseases, 67 (40% of total) approved drugs for Cardiovascular diseases, 46 (31% of total) approved metabolism drugs, 13 (30% of total) approved drugs for blood diseases and 32 (26% of total) approved antineoplastic drugs. Large percentage of these drugs in DRN shows that it may be a good way through DRN to get deep understanding of the mechanism of these drugs. Particularly, for metabolism drugs, we found that targets of the remaining are related to metabolism by their own specific protein function, but not by catalyzing metabolic reactions as an enzyme, thus this part of metabolism drugs are not involved in DRN. In order to illustrate which pathways the drugs interact with, we also colored metabolic reactions according to their pathways. The most frequent pathway in DRN is transport. 145 metabolic reactions of total 811 reactions in DRN are related to transport pathways. Note that in the BIGG database, there are six types of transport pathways: Lysosomal Transport, Mitochondrial Transport, Endoplasmic Reticular Transport, Extracellular Transport, Nuclear Transport and Golgi Apparatus Transport. For simplicity, we combine all these six transport pathways to one pathway as transport in Figure 3. The less frequent pathways are Carnitine shuttle (99), Keratan sulfate (44), Folate Metabolism (39), Tyrosine metabolism (33), Nucleotides (33) and Steroid Metabolism (30). Thus, Figure 3 illustrates not only the interactions between the cancer drugs and enzymatic reactions, but also the pathways each drug interacts with.

From Figure 3, we can see that the resulting network is naturally clustered by major therapeutic classes and major pathways, although DRN layout is generated without the knowledge of drug classes and reaction classes. The clustering of drugs may be mainly because they have the same targets. The most obvious cluster of drugs is the tightly connected drugs for the nervous system. Antineoplastic drugs, drugs for blood diseases, metabolic diseases, cardiovascular diseases, respiratory diseases and Musculoskeletal diseases are also clustered together, although less distinct than the cluster of drugs for nervous system. Reactions are clustered less obviously than drugs. The most obvious cluster of metabolic reactions is reactions related to folate metabolism and vitamin metabolism. Further observation from Figure 3 is the relationship between drug classes and reaction pathways. Most of the neurological drugs, blood drugs and cardiovascular drugs are connected with transport reactions. Many cancer drugs and some of cardiovascular drugs are related to the nucleotides reactions.

### Reaction profiling

**Figure 5:**
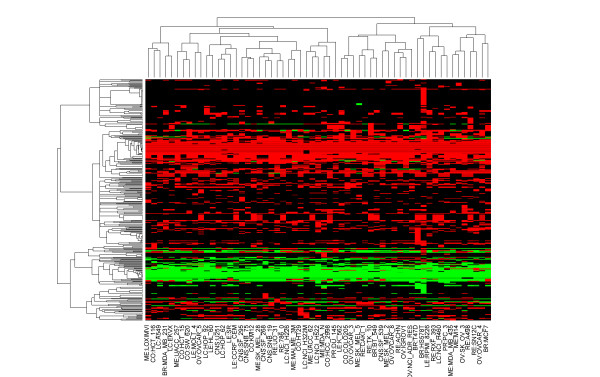
**Heat map of metabolic reaction profiles using complete linkage algorithm**. Rows represent different reactions, and columns represent different cell lines. Red and green indicate different directions of metabolic reactions, while black indicates a flux value very close to zero.

Reaction profiling we propose here is a method to quantify metabolic reactions. Using this method, the profile for each metabolic reaction is identified by its flux states in different cellular environments. Compared with the traditional equation representation of metabolic reaction, this new representation makes quantitative analysis of reactions obviously easier. In each of NCI-60 cell lines, LP model was used to predict metabolic flux distribution for all the metabolic reactions based on gene expression data in the cell line. Thus, each metabolic reaction was only designated one profile as its signature, which was used to quantitatively measure the similarity among reactions and thereby for prediction of novel drug-reaction interactions. We only focused on enzymatic reactions since only enzymatic reactions have direct interaction with drugs through enzymes. Around 2299 of total 3742 metabolic reactions in the human metabolic network can be catalyzed by enzymes. Some of the enzymes in metabolic network have been identified as drug targets. Among the 811 enzymatic reactions in DRN, the flux analysis shows that 400 reactions are inactive (-1 < flux < 1) in NCI-60 cancer cell lines. We only consider 353 reactions that are active in at least one cancer cell line. We report, in Figure 5, the heat map of these reaction profiles using cosine similarity and complete linkage algorithm. Red and green indicates different directions of metabolic reactions, while black indicates a flux value very close to zero. From the figure, we can see that both the enzymatic reactions and cancer cell lines cluster as two major groups. Especially, for enzymatic reactions, one group involves reactions with many positive fluxes (red), while the other group involves reactions with many negative fluxes (green). Note that very few differences between cell lines can be seen in some metabolic reactions, which may be due to the common biological properties of cancer cell lines, for example, uncontrolled growth, invasion, and sometimes metastasis. Although the two groups of cell lines are not as distinct as reactions due to these common properties, they may imply further study for these cancer cell lines.

**Figure 6:**
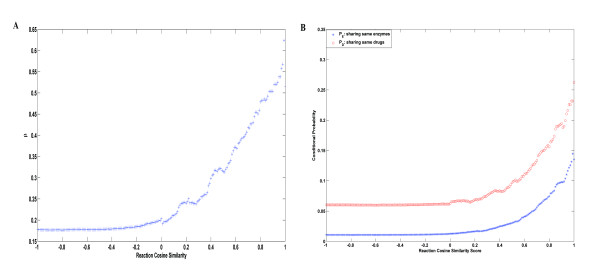
**Analysis for reaction flux similarity *s*_*RF*_**. 6A: the change of *β *as the cutoff value of reaction similarity changes; 6B: *P*_*E*_(*t*)and *P*_*D*_(*t*) are increasing functions of *t*.

To understand more about the relationship between reactions, enzyme targets, and drugs, we listed a table (Additional file 3) with reaction pairs in DRN that share the same associated drugs, their reaction flux similarity scores, their common enzymes, and their common associated drugs. Different reactions can interact with the same drugs in two ways. One is through the same enzymes, while the other is through different enzymes that are targets of the same drugs. Denote the set of the 353 considered reactions as *R*. The analysis shows that 3744 pairs of reactions Γ_*RDR *_share the same drugs while 670 pairs (Γ_*RER*_) share the same enzymes.  is defined to represent the percentage of reaction pairs that are associated with the same drugs through the same enzyme targets. *β *= 670/3744 = 0.18 indicates that around 18% reaction pairs interact with the same drugs because they are catalyzed by common enzyme targets, while the remaining 72% pairs interact with the same drugs through different enzyme targets. We also found that if reaction flux similarity is larger than 0.9, more than 50% of reactions interact with same drugs through the same enzymes. Figure 6A shows *β *increases as the cutoff value of reaction flux similarity increases. This means that if the reactions are more similar, they are more likely to interact with same drugs through same enzymes rather than through different enzyme targets.

Figure 6B illustrates how the probability of reactions interacting with the same enzymes/drugs changes with their similarity scores of reaction flux profiles. Although the maximum value of the probability is only around 0.4 for drugs and around 0.3 for enzymes due to the sparse relationship of drugs and reactions, it shows that reaction pairs with larger reaction flux similarity are more likely to interact with same drugs/enzymes. The observation implies that two reactions sharing high flux profile similarity tend to interact with same drugs/enzymes. One possible explanation is that, two highly similar reactions measured by reaction flux similarity may have functionally common properties in the context of cancer cell lines, thus the interaction of one with a drug may imply the interaction of the other with the drug. Therefore, for drug-reaction interaction prediction, it should be an effective way to depict the reaction space by reaction flux profile.

### Drug-Target interaction prediction by reaction flux similarity

We used two kinds of information for drug target prediction: 1) target similarity and 2) known drug-target interactions. Machine learning algorithms were first used to predict drug-reaction interactions, which then lead to novel drug-target interactions. The basic assumption is that if a drug is predicted to interact with a reaction, the enzyme catalyzing the reaction is likely to be the target of the drug. For the metabolic reactions, the constraint-based flux analysis in NCI-60 cell lines were first applied to obtain reaction profiles. Reaction flux similarity is then defined as the cosine similarity among reaction flux vectors. All the known drug-reaction interactions are obtained from the DrugBank database. Since we represent each metabolic reaction based on NCI-60 cancer cell lines, we focused on 32 cancer drugs in DRN to perform cross validation of the prediction method. We eliminated the reactions that have inactive flux states (-1 < flux < 1) in all the 59 cell lines. Training set includes 260 metabolic reactions. We denote this dataset as SET-I, which contains 32 cancer drugs, 260 metabolic reactions in DRN and their ground truth interactions. We also generated the dataset, SET-II, which includes 32 cancer drugs, available expression in NCI-60 for genes encoding 629 targets and their ground truth interactions. SET-I was used to predict drug targets through target reaction classification based on target reaction similarity, while SET-II was through target gene classification based on target gene similarity. In the process of classification, both proteins and reactions are considered as drug targets.

Different classification methods were performed on both SET-I and SET-II, such as Nearest Profile, Bipartite graph model [8], Support Vector Machine (SVM), K-Nearest Neighbor (KNN) and Kernel KNN. Nearest Profile simply assigns new target to the drug class labels of its nearest neighbor. Bipartite graph model first measures the similarity among all nodes in the bipartite network by their shortest paths, then maps all the nodes into a common space preserving the distances among all nodes. A new reaction target is mapped to the common space first, and then correlation with all drugs is calculated. Large correlation indicates high probability of drug-target interaction. For SVM, we used a Radial Basis Function (RBF) as kernel.

Table 1 shows the prediction results of drug-target interaction. Mean AUCs (Area Under Curves) for 50 times are reported for each method and each dataset. From the table we can see that the performance of drug target prediction through target reaction similarity is much better than through target gene similarity for almost all classification approaches (Note that when using the KNN classification approach, the performances of two methods are similarly bad). This implies that the metabolic network provides important and useful information for the drug target prediction. It also indicates that target reactions are easier to be classified than target genes. Among all the target reaction classification methods we used, Kernel KNN performs best. Its AUC can reach 0.85. The second best classification method is SVM, AUC of which can reach 0.81. One possible reason to explain why Kernel KNN is better than SVM is that the interactions between drug and targets are very sparse, which can cause the relatively bad performance of SVM. Bipartite graph model performs badly due to the sparse interactions between drugs and targets. The result also proves that reaction profiling is an effective approach to quantify metabolic reactions, and the similarity measured in this way is meaningful for drug target prediction.

**Table 1:** Drug target Prediction Performance

AUC	Nearest Profile	KNN	Bipartite	SVM-RBF	Kernel KNN
**Target Gene Classification**	0.6267	0.6570	0.4994	0.7791	0.6263
**Target Reaction Classification**	0.6911	0.6589	0.5276	0.8136	0.8469

### Integration with target sequence data

**Figure 7:**
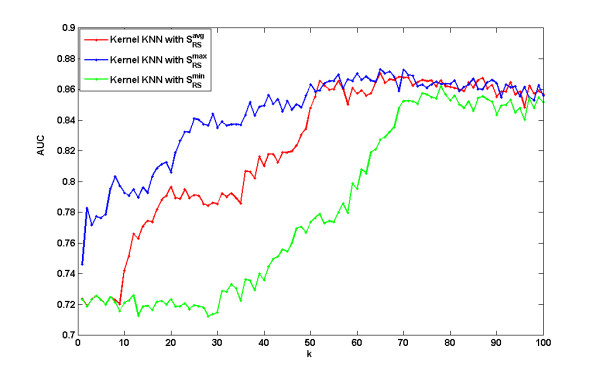
**Comparison of the kernel KNN method with different reaction structure similarity s^max^_*RS*_, s^*avg*^_*RS *_and s^min^_*RS*_**. The three curves show how the corresponding prediction AUCs change when the parameter *k *changes.

**Figure 8:**
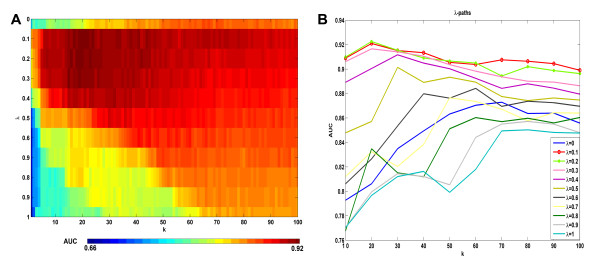
**The prediction performance by the integration of reaction profiles with target sequence**. 8A: The heat map of the mean AUCs at all considered *λ *and *k*. 8B: The *λ*-paths of the heat map.

In Figure 7 we report the prediction performances of the three reaction structure similarities  and  using Kernel KNN method. We can see that among the three similarity measurements,  performs the best. We then further evaluated the prediction performance by integrating reaction flux profiles with target sequence. Figure 8 reports the prediction accuracy of Kernel KNN method using the integrated reaction similarity s_*R *_= *λs*_*RF *_+ (1 - *λ*)s_*RS*_, where . We performed 50 times 10-fold cross validation of Kernel KNN for each combination of *k *= 1 ~ 100 and λ = 0,0.1,0.2,...,1, where *k *is the parameter in Kernel KNN method and *λ *is the weighting of reaction flux similarity mentioned in the section of Methods. Figure 8A is a heat map for all the mean AUCs by corresponding *k *and *λ*. It shows that the maximum prediction accuracy is obtained around *k *= 20 and *λ =*0.1 ~ 0.3. In fact, the best AUC can reach 0.92 and the worst AUC is around 0.66. Figure 8B further plots the *λ*-paths of the heat map. When *λ *= 0, only enzyme target sequence is used for classification, while when *λ *= 1, only reaction flux profile is used. From the figure of *λ*-paths we can see that *λ*-paths for *λ *= 0.1 ~ 0.6 are almost always higher than *λ*-path for *λ *= 0, this means the integration of reaction flux with target sequence makes the classification performance better, for almost all *k *= 10 ~ 100. We also performed the t-test on the respective 50 AUCs of *λ *= 0 and *λ *= 0.2 when *k *= 20 is chosen, the resulting p-value of 1.17 e-028 shows the difference is statistically significant. The synergism of reaction flux profiles and enzyme target sequences further supports that reaction flux similarity obtained by reaction profiling does help for exploring the associations between drugs and reactions or targets. Figure 8 shows that *λ *= 0.1 ~ 0.3 are best choice for the classification model. From the figure of *λ*-paths of the prediction performance, we can also see that the changes of the performance by *k *at around *λ *= 0.1 ~ 0.3 is much less than *λ *= 0 and *λ *= 1, this means the prediction becomes more stable after the integration of two data sources.

We also further compare the prediction results of the methods using *λ *= 0 and *λ *= 0.2 by removing metabolic reactions one after another from the training data and then predicting its associations with all the drugs. This is the Leave-One-Out method. We chose *k *= 20 for the validation. The prediction AUC by the methods using *λ *= 0 and *λ *= 0.2 is 0.83 and 0.85, respectively. This shows that the integration of flux information can improve the prediction accuracy. For each reaction, we further reported in Additional file 4 its related drugs with association score larger than the cutoff value 0.35. There are 24 and 27 predicted drug reaction pairs for the methods with *λ *= 0 and *λ *= 0.2, respectively. Although the two methods have 13 common predicted drug-reaction pairs, with 11 true positives and 2 false positives, their results have some differences. By the method with *λ *= 0.2, we can get 12 other true positives and only 2 false positives, while with *λ *= 0 we can get 3 other true positives and 8 false positives. Specifically, the method with reaction structure similarity cannot identify the targets AMPD1, AMPD2, AMPD3, GMPR, GMPR2, GMPS, HPRT1, IMPH1 and IMPH2 of azathioprine and mercaptopurine, and the target ABCC1 of daunorubicin, while the integration of the reaction flux similarity can identify the relationships by leveling up the their association scores. For example, ABCC1, the multidrug resistance protein 1(also known as MPR1), is one of the approved targets [21-23] of daunorubicin. It can be re-identified if we integrate reaction flux information and reaction enzyme structure information. Belonging to the ATP-binding cassette (ABC) superfamily of transport proteins, ABCC1 can confer resistance to daunorubicin in human tumor cells. MRP1 is expressed in normal tissues acting as an efflux pump for glutathione, glucuronate, and sulfate conjugates and may thus influence the pharmacokinetic properties of many drugs. The intracellular accumulation of daunorubicin is decreased and the efflux of daunorubicin is increased in the transfectant. Furthermore, the reaction flux similarity can also avoid the false positives produced by reaction structure similarity by depressing corresponding association scores.

**Figure 9:**
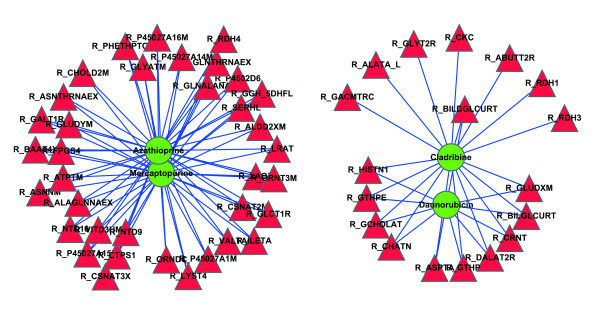
**Top 100 predicted related reactions for cancer drugs**. Red and green nodes represent reactions and drugs, respectively.

We then predict new reactions and enzyme targets for the cancer drugs by Kernel KNN of *k *= 20 and *λ *= 0.2. The results show that for four cancer drugs Daunorubicin, Cladribine, Mercaptopurine and Azathioprine, some new related reactions are identified with high association scores. We report all the predicted reactions and enzymes for these 4 drugs in Additional file 5. Figure 9 shows these new related reactions for the four cancer drugs. 36 reactions are predicted to be associated with both Mercaptopurine and Azathioprine, other 11 reactions are for both Daunorubicin and Cladribine, and the remaining 8 reactions are for only Cladribine. It is reasonable that Mercaptopurine and Azathioprine are predicted to interact with exactly same reactions since the two drugs have the same known targets. These predictions may lead to further biological research.

Daunorubicin is an aminoglycoside of the anthracycline class that is mostly used in the treatment of leukemia. The cytotoxic activity of daunorubicin is through intercalation between DNA base pairs, which results in DNA strand breakage. The drug also inhibits topoisomerase II and polymerase. Daunorubicin is the substrate of ATP-binding cassette proteins (sub-family C member 1 and sub-family B member 1). Studies indicate that daunorubicin is effluxed through these transporters together with glutathione[24]. The stoichiometry of the co-transport is approximately 1:1. Cancer cells typically overexpress multidrug resistance proteins and also have high levels of glutathione. Glutathione confers survival advantage to cancer cells not only by increased resistance to oxidative stress and apoptosis, but potentially also by increased efflux of anticancer drugs. The other three identified glutathione peroxidases (GPX1, GPX2 and GPX4) all catalyze the reduction of reactive oxygen species by reduced glutathione, and therefore protecting cancer cells from oxidative damage. Studies have demonstrated that chemotherapy leads to a decrease of glutathione peroxidase activity. The prediction of the glutathione peroxidases as potential targets of daunorubicin provides a plausible explanation of this phenomenon.

Cladribine is a synthesized analog of the nucleoside adenosine. It interferes with synthesis and repair of DNA by inhibiting adenosine deaminase, and therefore has a strong inhibitory effect on the rapidly-replicating leukemia cells. The *in vivo *phosphorylated derivative of cladribine is incorporated into DNA and leads to DNA strand breakage. Cladribine and some of its analogs have also been shown to be substrates and inhibitors of *E. coli*. purine-nucleoside phosphorylase, although they do not react with mammalian purine nucleoside phosphrylase [25]. Some analogs, however, are active against leukemia cells [25]. The GPX peroxidases are also predicted as targets of cladribine. It is likely that the drug may also inhibit these enzymes, weakening their protective role in leukemia cells.

Mercaptopurine is a purine analogue which interferes with nucleic acid biosynthesis by inhibiting purine metabolism and has been found active against human leukemias. It is usually used in the treatment of or in remission maintenance programs for leukemia. Our prediction suggests that the mechanism of action of mercaptopurine may be related to 5'-nucleotidase (GMP). This reaction preferentially hydrolyzes inosine 5-prime-monophosphate (IMP) and other purine nucleotides, and is allosterically activated by various compounds, including ATP. The enzyme GMP, also known as NT5C2, is exclusively located in the cytoplasmic matrix of cells and may have a critical role in the maintenance of a constant composition of intracellular purine/pyrimidine nucleotides in cooperation with other nucleotidases. Besides NT5C2, GGH and FPGS are also suggested to be targets of mercaptopurine. These two enzymes are both in the pathway of folate biosynthesis. Folic acid is especially important during periods of rapid cell division and cell growth. Mercaptopurine may inhibit the tumour cell growth by interfering folate biosynthesis. SLC25A15 and SLC25A2, two orinithine transporters for the urea cycle in liver, are also predicted as targets of mercaptopurine. The two transporters are responsible for removing ammonia from blood. Besides the predicted reactions and targets listed in Table 2, Table S2 in Additional file 5 lists other possible targets. Enzyme CTPS for CTP synthase (NH3), and NT5C2 for 5'-nucleotidase (XMP) are possible targets. Although they have not been approved as targets of mercaptopurine, these reactions and related pathways are known to act closely to mercaptopurine. For example, some mercaptopurine is converted to nucleotide derivatives of 6-thioguanine (6-TG) by the sequential actions of inosinate (IMP) dehydrogenase and xanthylate (XMP) aminase, converting TIMP to thioguanylic acid (TGMP). Besides, the reaction xanthine oxidase with its Enzyme XDH is also a promising target since studies [26,27] have shown that mercaptopurine is inactivated by xanthine oxidase.

**Table 2:** Top Predicted Reactions for 4 drugs.

Drug Name	Reaction Name	Sub System	Target Enzyme	Score
Cladribine	Glutathione peridoxase	Glutathione Metabolism	GPX1/GPX2/GPX4	0.6145

Daunorubicin	Glutathione peridoxase	Glutathione Metabolism	GPX1/GPX2/GPX4	0.5631

Azathioprine/Mercaptopurine	Gamma-glutamyl hydrolase (5DHF), lysosomal	Folate Metabolism	GGH	0.5692
	Ornithine mitochondrial transport via proton antiport	Transport, Mitochondrial	SLC25A15/SLC25A2	0.5598
	5'-nucleotidase (GMP)	Nucleotides	NT5C2	0.5477
	Folylpolyglutamate synthetase (DHF)	Folate Metabolism	FPGS	0.545

Like mercaptopurine, azathioprine is also an immunosuppressive agent which works by suppressing the body's immune response. Azathioprine is a prodrug which is converted to 6-methyl-mercaptopurine by thiopurine methyl transferase (TPMT) and then metabolised to the active metabolite 6-thioguanine. Thus it's reasonable to predict the same reactions/targets for mercaptiopurine and azathioprine.

The activity of these four cancer drugs against the predicted enzymes can be tested by direct enzyme activity assays. Considering some drugs may need *in vivo *activation (e.g., cladribine), enzyme activity is best measured after administration of the drugs both *in vitro *and *in vivo *(e.g., in cell cultures or animal models). If there are known mutations that affect enzyme structure or activity, these may be taken advantage of to see if the mutations also alter effect of the predicted drugs on the enzyme.

## Conclusions

In this study, we first construct a drug reaction network to show their relationships in a unified context, and then present a reaction profiling method to depict systematically the metabolic reactions in a quantitative way. Reaction flux profiling makes it possible to measure the similarity of metabolic reactions functionally, which is a key step in many kinds of data analysis techniques such as clustering and classification. The comparison of target gene classification with target reaction classification shows the effectiveness of the indirect method to predict drug targets, and implies the important contribution of reaction flux similarity in drug target prediction. We also integrate the reaction structure similarity with this reaction flux similarity, and the results show the synergism of the structural similarity and functional similarity. We also notice that the integration of two similarity metrics makes the prediction more stable. This further implies the effectiveness of reaction profiling for drug target prediction. We thus predict some related reactions and possible targets for four cancer drugs for further biological research.

Note that one limitation of reaction profiling lies in that proteomics and phorsphoproteomics are not involved. It may decrease to some extent the impact in the understanding of the multitude of interactions between drugs and entire metabolic network. In the future work we will address it when proteomics and phorsphoproteomics data become available. Metabolic reaction profiling has more applications than what we have discussed in this work. Besides NCI-60 cancer cell lines, other disease cell lines can be used to discuss the related metabolic reactions. The statistics of the drugs in DRN show that drugs for metabolic diseases, cardiovascular diseases or antineoplastic drugs are the most related to metabolic networks. This gives us a hint that these three diseases may also be discussed using metabolic network. We also note that our flux analysis is based on the mRNA content in the context of transcription, thus further flux analysis using metabolomic data may be developed to explore the underlying mechanism of interactions between drugs and metabolic reactions. Another possible application is metabolic biomarker discovery, which attempts to explore significant metabolites, metabolic reactions and enzymes. A biomarker discovery method involving gene expression and protein-protein interaction has been developed by Ideker et al. [28]. It compares the gene expression over particular subset of conditions to identify active sub networks. This idea can be integrated with reaction flux profiling to identify significant metabolic reactions. We can first predict the metabolic states of cells before and after the absorption of drugs based on the gene expression data, and then compare the reaction flux over different conditions to identify significant metabolic reactions, from which the related metabolites biomarkers can also be identified.

## Authors' contributions

LL and XZ conceived the idea and designed the research. LL acquired and processed the data. LL performed the research and analyzed the results. LL, XZ and WC wrote the paper. All authors read and approved the final manuscript.

## Supplementary Material

Additional file 1 **Different reaction measure of similarity**. The file gives the comparison of performance using different reaction measure of similarity in Kernel KNN Click here for file

Additional file 2 **Drug-Reaction Network**. This file lists the detailed information in Drug-Reaction Network Click here for file

Additional file 3 **Reaction flux similarity score **This file lists the reaction flux similarity and the common drugs between each pair of metabolic reactions. Click here for file

Additional file 4 **The performance for the integration of reaction flux similarity and reaction structure similarity**. This file lists the performance for the integration of reaction flux similarity and reaction structure similarity. Click here for file

Additional file 5 **The predicted reactions and targets for cancer drugs**. This file lists the predicted reactions and targets for cancer drugs. Click here for file
