# Supplementary material for: Predicting enzyme targets for cancer drugs by profiling human Metabolic reactions in NCI-60 cell lines
Source: BMC Bioinformatics. 2010 Oct 8;11:501. doi: 10.1186/1471-2105-11-501 (PMC2964682; doi:10.1186/1471-2105-11-501)
Supplement: Additional file 1 — Different reaction measure of similarity. The file gives the comparison of performance using different reaction measure of similarity in Kernel KNN [file 1471-2105-11-501-S1.DOC]

**Supplementary Material**

**Predicting Enzyme Targets for Cancer Drugs by Profiling Human Metabolic Reactions in NCI-60 Cell lines**

**Reaction Flux Similarity**

After we get the profiles for metabolic reactions, we need to measure their similarities so as to establish the reaction space. The results reported in the manuscript are all based on cosine similarity of reaction profiles. We now compare the prediction performance using cosine similarity of target reaction profiles and their similarity defined by the Euclidean distance. Figure 7S shows how AUC changes after changing $k$ in Kernel KNN method. It indicates that although the prediction performance remains unchanged when $k$ increases, cosine similarity is always better than the similarity defined by Euclidean distance. Figure 7S also shows that for Kernel KNN method, AUC tends to be stable when $k$ increases. The reason is that when $k$ increases, a metabolic reaction’s new neighbors contribute very few due to their low similarity with the considered reaction.


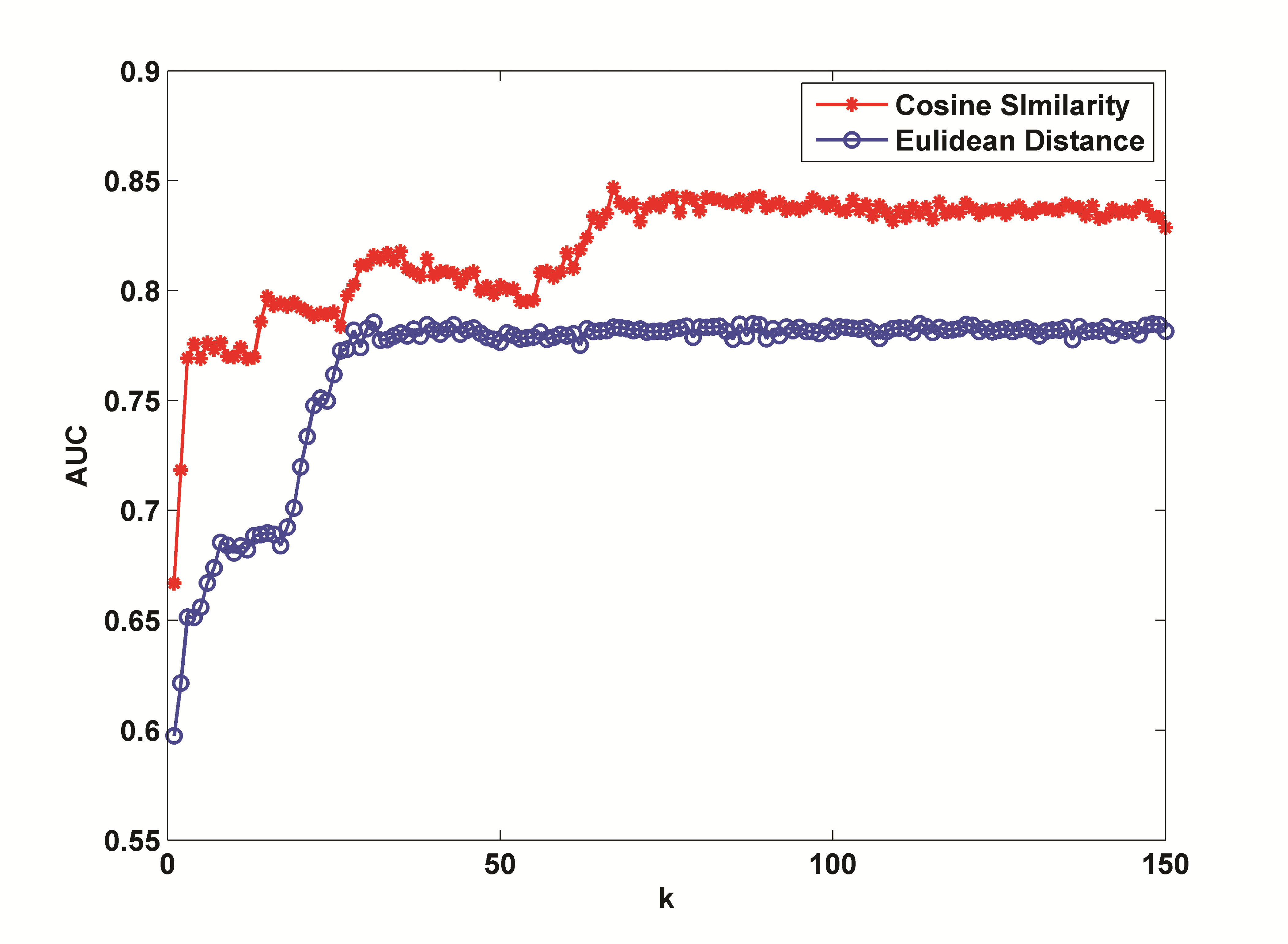


**Figure S1:** Comparison of performance using different measure of similarity in Kernel KNN. Red (blue) solid line represents the change of AUC as the number of neighborhood $k$ changes using cosine similarity (Euclidean distance).
